# A Rare Case of Measles-Associated Hemophagocytic Lymphohistiocytosis in an Infant

**DOI:** 10.7759/cureus.8246

**Published:** 2020-05-23

**Authors:** Theano Lagousi, Paraskevi Korovessi, Eleni Panagouli, Vasilis Tsagris, Stavroula Kostaridou

**Affiliations:** 1 Paediatrics, Penteli Children's Hospital, Athens, GRC

**Keywords:** measles, secondary hemophagocytic lymphohistiocytosis, immunoglobulin, vaccination

## Abstract

Measles continues to be a threat in most European countries due to suboptimum vaccination coverage. Although measles leads to several complications, measles-related hemophagocytic lymphohistiocytosis (HLH) has been rarely reported. Herein, we present a case of a four-month-old male infant, the first child of unrelated, healthy parents, with no significant medical history or unexplained infant death in the family, otherwise healthy, who was diagnosed with measles-associated HLH and was successfully treated with IV dexamethasone and IV immunoglobulin (IVIG). Additionally, we review previously reported cases of HLH secondary to measles and highlight the diagnostic and therapeutic challenges associated with its early recognition and treatment. High suspicion, early recognition, and appropriate treatment are essential for a favorable outcome of measles-associated HLH.

## Introduction

A large measles epidemic has affected European countries in the past three years [1]. During this period, Greece saw the second largest measles outbreak in Europe [1]. In 2017, based on the local surveillance data from the Hellenic Center for Disease Control & Prevention, 968 cases were recorded, after three years without local endemic measles transmission, starting from a cluster of three imported cases in Northern Greece, concerning unvaccinated Romanian Roma siblings [2]. The outbreak has spread all over Greece, reaching 3150 reported cases, primarily in the southwestern country. Most cases were of Roma origin, especially children <10-year-old, followed by Greek nationals, mostly young adults, not immune to measles and some nonvaccinated healthcare professionals (HCPs) [2]. Overall, four deaths have been reported. Broad vaccination campaigns that were implemented afterward in refugee/migrant hosting sites prevented the extensive spread of measles in such populations [2]. Currently, further efforts focus on raising awareness among HCPs and on organizing vaccination campaigns in hard-to-reach vulnerable populations such as the Roma population where vaccination rates remain low.

 Hemophagocytic lymphohistiocytosis (HLH) is a rare and occasionally fatal disorder characterized by abnormal proliferation of macrophages, hypercytokinemia, and T-cell immunosuppression leading to multiorgan failure [3-4].

Hemophagocytic lymphohistiocytosis may be familial, affecting infants (fHLH), or occur at any age secondary to infection, malignancy, or rheumatologic disease (sHLH) [5]. In 2001 the Histocyte Society revised the diagnostic criteria and therapeutic approach for HLH (HLH-2004-protocol) [5]. For the diagnosis, five of the eight criteria [1:fever, 2:splenomegaly, 3:cytopenias at least in two blood cell-lines, 4:hypertriglyceridemia and/or hypofibrinogenemia, 5:hemophagocytosis in bone marrow, spleen, or lymph nodes, 6:low or absent natural killer (NK)-cell activity, 7:hyperferritinemia, 8:high levels of soluble-IL2r] must be fulfilled. Other clinical and laboratory findings supporting HLH-diagnosis include cerebrospinal-fluid pleocytosis and/or elevated spinal fluid protein, histologically confirmed chronic hepatitis, cerebromeningeal symptoms, lymphadenopathy, jaundice, edema, rash, transaminitis, hypoproteinemia, hyponatremia, hyperbilirubinemia, and serum lactate dehydrogenase (LDH)>1.000 IU/L [5-6].

Although measles is associated with high morbidity and mortality rates due to several complications, measles-induced HLH has been rarely described. Herein, we describe a case of an infant with potentially life-threatening measles due to associated HLH. Additionally, we review reported cases of measles-associated HLH and highlight the diagnostic and therapeutic challenges associated with its early recognition and treatment.

## Case presentation

**Figure 1:**
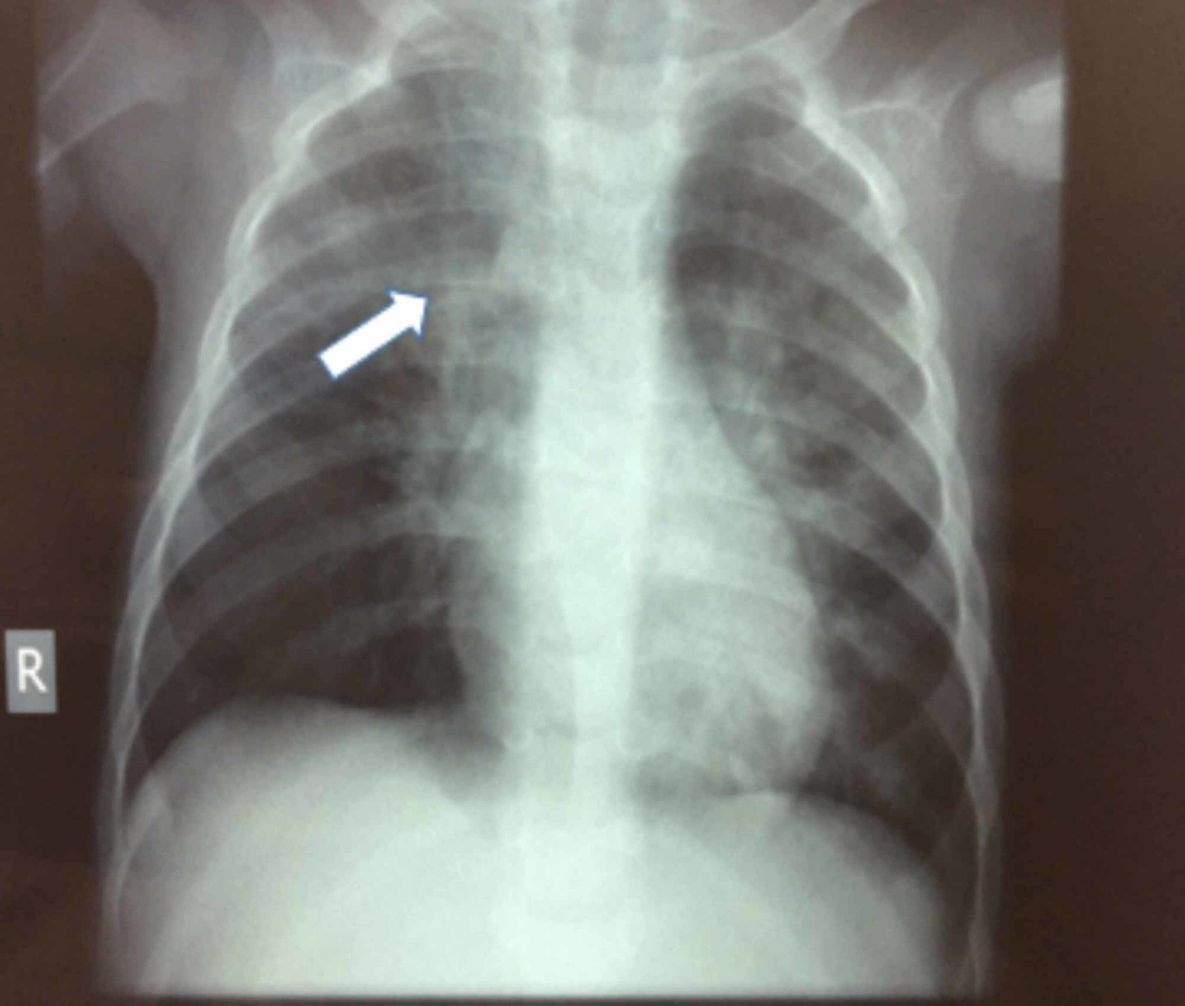
Patient's chest X-ray. Chest X-ray in admission showing mild infiltration

A four-month-old male infant, the first child of unrelated, healthy parents, with no significant medical history or unexplained infant death in the family, otherwise healthy, presented with severe respiratory distress due to measles-associated pneumonitis (nine days after the disease onset). His initial physical examination revealed a febrile in poor general condition, with a respiratory rate at 70/min, oxygen saturation at 93%-94%, and diffuse crackles and wheezing. He had a generalized erythematous maculopapular rash, while Koplik spots were not visible. There was no conjunctivitis, lymphadenopathy, or hepatosplenomegaly. Admission laboratory-tests including full-blood-count, C-reactive protein (CRP), procalcitonin, kidney/liver function, and serum electrolytes were normal. Measles IgM antibodies were detected serologically. Initial and repeated blood-cultures did not yield any pathogen. Abdominal ultrasound in admission was normal, while chest X-ray presented only mild inflitrations and no signs of pneumonia (Figure 1). He was met on nebulized racemic epinephrine and oxygen administration, and was started on IV cefotaxime due to a potential concomitant bacterial co-infection, suspecting sepsis due to affected clinical condition.

**Figure 2:**
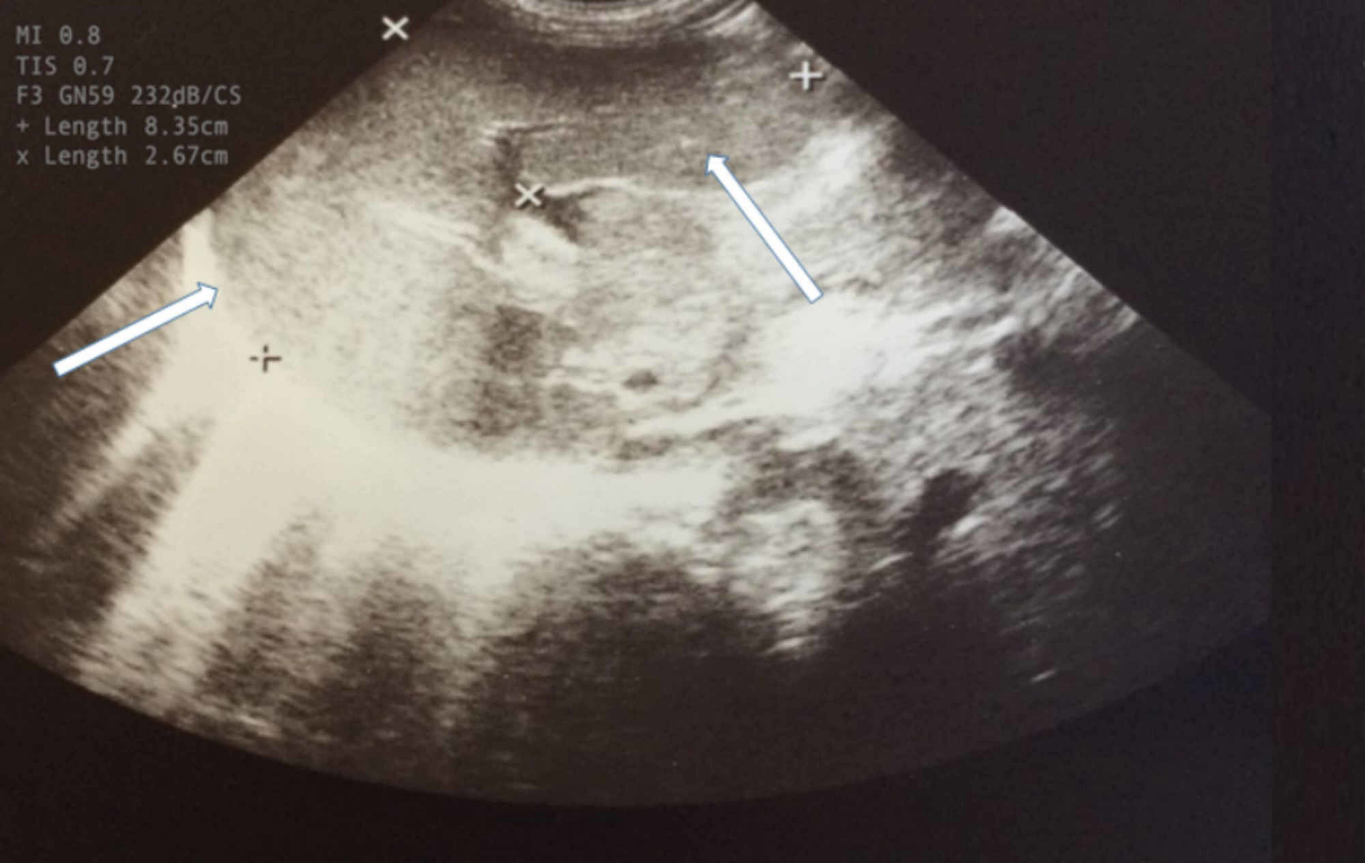
Patient's ultrasound. The second (repetitive) ultrasound showed splenomegaly (8.35 cm)

Five days later, he was still in poor general condition with persistent respiratory distress and fever, while hepatosplenomegaly was revealed in the new ultrasound (Figure 2).

**Figure 3:**
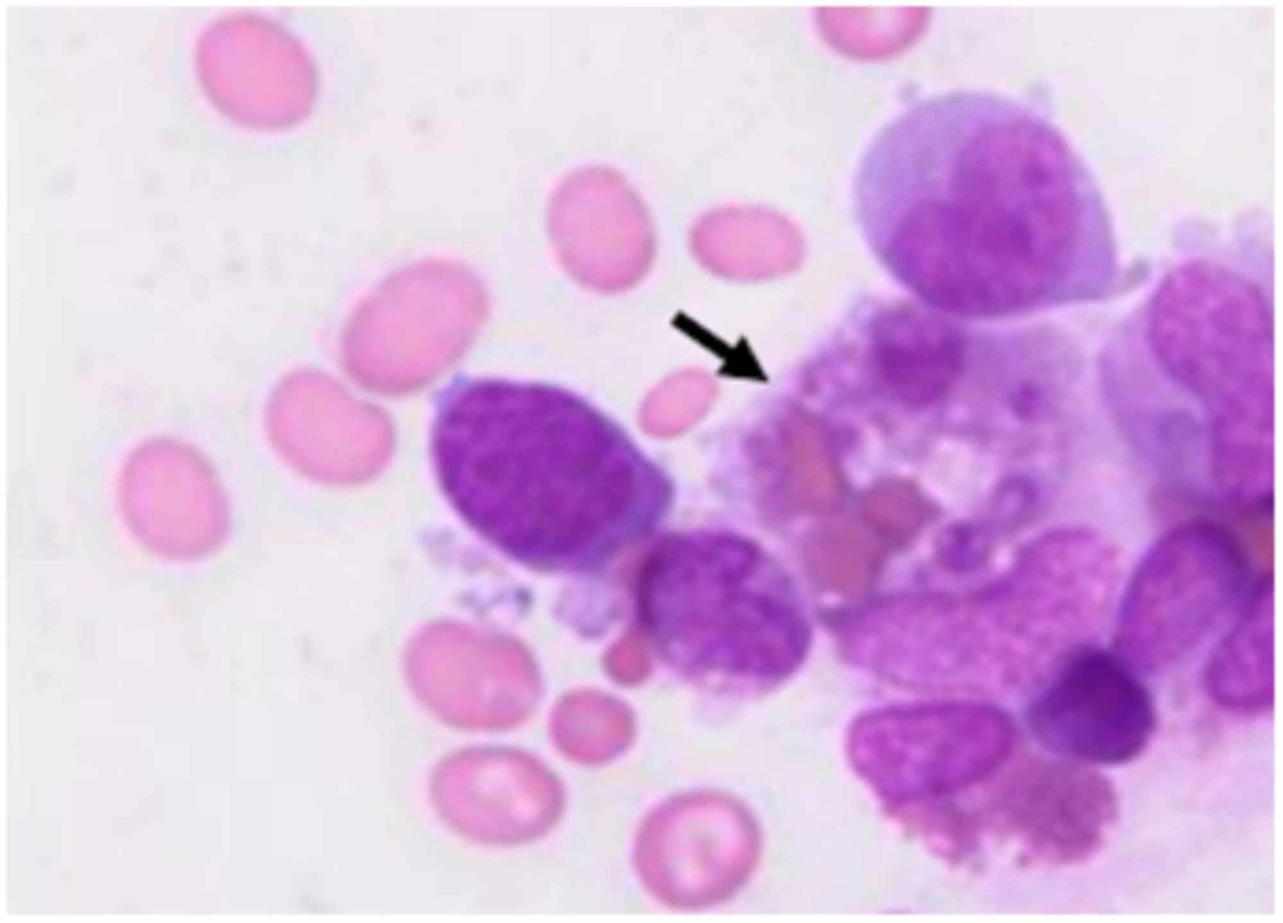
Periodic acid-Schiff staining of the bone marrow aspirate. The black arrow indicates a macrophage which phagocytized an immature red blood cell

Further investigations revealed substantially elevated serum ferritin (~10000 μg/L) and slight hypofibrinogenemia (1.34 g/L). All the biochemical exams performed in the patient are presented in Table 1. As HLH was considered, bone marrow (BM) aspiration was performed, showing hemophagocytosis without blasts (Figure 3).

**Table 1:** Biochemical tests during patient’s hospitalization. WBC: white blood cells, HgB: hemoglobin, PLT: platelets, APPT: activated partial thromboplastin time, SGOT: serum glutamic oxaloacetic transaminase, SGPT: serum glutamic pyruvic transaminase, LDH: lactate dehydrogenase, TGL: triglycerides. Normal range is also depicted (last column). §: laboratory test values fulfilling HLH-2004-criteria. *: supportive HLH-2004-criteria fulfilled when HLH diagnosis was set.

Laboratory tests	Hospitalization day									
	2	3	4	5	6	7	8	9	10	Normal range
WBC (X10^3^/mm^3^)	16.4	20.6	11	12.5	4.09	5.72	10	14.1	15.2	6-17.5
Neutrophils (10^3^/mm3)	2	3.5	2.7	2.1	1.9	1.5	0.7^§^	2.1	4.2	1-8.5
HgB (g/dL)	13.6	13.1	13	13.2	12	11.4	10.3	8.2^§^	9.9	9.5-14.1
PLT (X10^3^/mm^3^)	372	351	269	252	190	123	85^§^	135	160	150-350
SGOT (IU/L)	56	103	379	454^*^	280	204	161	56	49	15-60
SGPT (IU/L)	33	42	172	216^*^	174	192	271	150	106	13-45
Serum albumin (g/dL)				3.7	3.1	3.3			3.9	3.4-5.4
Sodium (mEq/L)	142	139		137		140		139	135	135-145
Ferritin (μg/L)			7230	9943^§^	6400	4515	2538	1163	600	50-200
TGL (mg/dL)			140	168	245	592^§^	575	297	181	30-86
LDH (IU/L)				1540^*^	971	519	367	252	242	180-430
APTT (s)				47.1		30.	32.4	26.5		24-36
Fibrinogen (g/L)				1.34^§^		1.9				1.7-4.05
D-Dimers (μg/mL)				3.5^*^		1.6				<0.5

**Figure 4:**
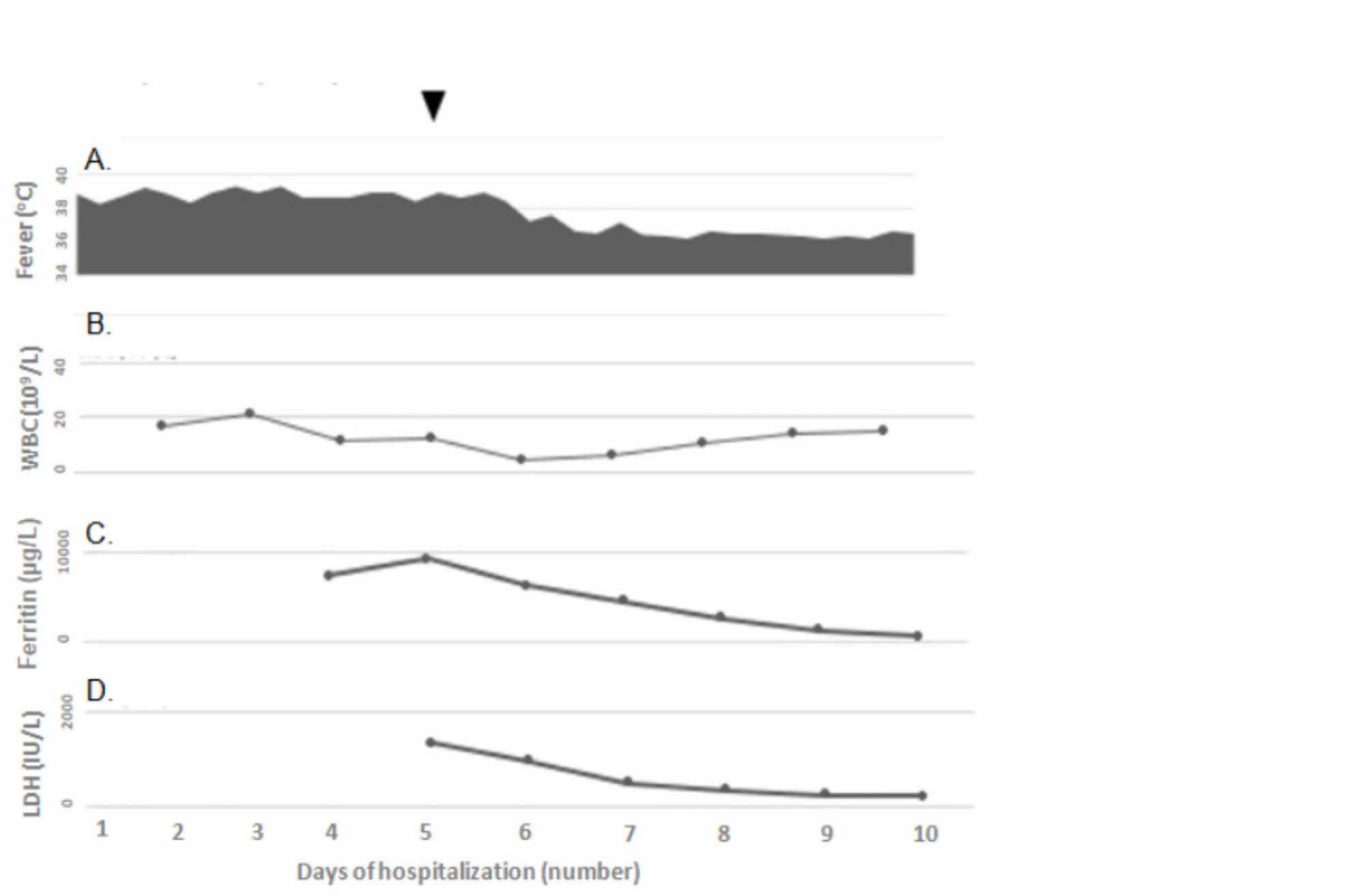
The clinical course of the patient. The arrow indicates the onset of the treatment with dexamethasone and intravenous immunoglobulin (IVIG). The numbers on the horizontal axis represent the days of hospitalization. A. Body temperature. B,C,D: Laboratory changes: White blood cells (WBC), Ferritin, Lactate dehydrogenase (LDH) respectively. Ferritin and LDH were not asked before Day 4-5 when hemophagocytic lymphohistiocytosis (HLH) was suspected.

Our patient fulfilled five out of eight criteria of HLH-2004-criteria; fever, splenomegaly, hyperferritinemia, hypofibrinogenemia, and ΒΜ hemophagocytosis. Transaminitis (aspartate transaminase 454 IU/L and alanine transaminase 216 IU/L) and increased D-dimers (3.5 μg/mL) and lactate dehydrogenase (1540 IU/L) further supported HLH-diagnosis. Hypertriglyceridemia (reaching 592 mg/dL) and a drop in all blood cell-lines (reaching the following values: neutrophils 0.7 x 109/L, hemoglobin 82 g/L, and platelets 85 x 109/L) presented later in the disease course. As there was no diagnostic uncertainty based on the above criteria, NK function or CD25 levels were not measured. The most common triggering causes of sHLH including infectious agents (EBV, CMV, parvovirus B19, enterovirus, adenovirus, influenzae virus, and leishmania) and basic rheumatologic investigations (antinuclear/anti-double stranded DNA antibodies) were all negative. Total subclasses of immunoglobulins and peripheral blood immunophenotype were normal. Therefore, our patient was diagnosed as having measles-associated sHLH and was managed with IV dexamethasone and IV immunoglobulin (IVIG), and supportive chemoprophylaxis. He progressively recovered, staying afebrile from the first week of treatment onset. Dexamethasone was continued for eight weeks and the patient remained well. No signs of relapse occurred 15 months later. The clinical course and the progression of the patient’s laboratory parameters are shown in Figure 4.

## Discussion

While fHLH is caused by mutations in genes involved in perforin-dependent lymphocyte cytotoxicity, sHLH is associated with infection, malignancy, or rheumatic conditions [3-4]. It is often difficult to distinguish the two HLH forms. sHLH is our most likely diagnosis due to the absence of a positive family history, parental consanguinity, and familiar history of unexplained pediatric death. Our patient rapidly responded to the initial treatment with a long remission state, further reducing the possibility of fHLH, that is often more severe, associated with frequent relapses and higher mortality rates. Fever, splenomegaly, hyperferritinemia, cytopenias associated with respiratory distress, and/or multiorgan failure should prompt a strong consideration of sHLH [5]. Remarkably, although blood cell-lines were within the normal range, it is the drop from previous levels that may herald HLH development [5]. fHLH cannot be excluded considering the early-life onset of HLH as well as previous reports showing that infectious agents may trigger fHLH. Therefore, molecular investigation of fHLH mutations and/or NK-function and soluble-CD25 assessment may be recommended, especially in the context of a possible future reactivation.

Measles-induced sHLH has been previously reported in five cases (Table 2). All cases presented with pneumonitis [7-11], while only three had also cerebromeningeal symptoms [7, 9]. Three cases occurred in immunocompetent children [8, 10-11]. Among the adults, there was an immunocompetent 18-year-old man with acute respiratory distress [10] and a 17-year-old boy with high risk acute lymphoblastic leukemia [11]. Death occurred in a previously healthy 25-month-old boy with hemophagocytic syndrome, and acute disseminated demyelinating encephalitis and a 17-year-old boy with high-risk acute lymphoblastic leukemia [9, 11]. To our knowledge, this is the first case of measles-associated sHLH in an infant.

**Table 2:** Previously reported cases of measles-induced hemophagocytic lymphohistiocytosis (HLH).

Reference	Yamamoto et al. [7]	Joshi et al. [8]	Pearl et al. [9]	Komatsuda et al. [10]	Huang et al. [11]
Gender	Male	Female	Male	Male	Male
Age (years)	8	School-aged girl	2	18	17
Immunocompromised	No	No	No	no	Yes (acute lymphoblastic leukemia)
Treatment	Cyclosporine A-methylprednisolone	Supportive treatment	VP-16, an epipodophyllotoxin	Methylprednisolone	Steroids/etoposide/plasma exchange
Clinical outcome	Favorable	Favorable	Died	Favorable	Died

The molecular mechanism involved in measles-induced HLH is not yet defined. CD150/signaling-lymphocytic-activation-molecule, a T/B/dendritic cell glycoprotein, is a costimulatory receptor involved in T-cell activation and a cellular receptor for measles virus [12-13]. Considering that CD150-induced signal transduction is controlled by SAP/SH2D1A gene that is aberrant in X-linked lymphoproliferative disease and fHLH, measles virus may affect CD150 function in a way similar to genetic disorder leading to transient excessive macrophages, NK cells and lymphocytes activation, leading in turn to excessive immunoactivation, characterizing sHLH. Therefore, in cases of measles-induced HLH, where a genetic disorder is suspected, SAP/SH2D1A mutation should be investigated; albeit, further research is required to verify this assumption.

Finally, another issue worth mentioning is that measles-induced sHLH management was quite challenging. Firstly, the use of steroids in viral-induced sHLH has been widely debatable. Although, most studies reported no impact on the course of disease progression, a single one described reversal of cytopenia and respiratory failure with pulsed steroids [10]. Therefore, we opted to initiate treatment with immunomodulatory agents (high-dose corticosteroids and IVIG) rather than the standard HLH-2004-protocol, to balance immunosuppression, ready to immediately switch to HLH-2004-protocol at the first sign of failure, something that was not required due to our patient’s acute response. This is in accordance with the previous reports supporting the use of IVIG and/or corticosteroids for the first-line treatment of virus-induced sHLH [14-15]. Such an approach may avoid etoposide-induced toxicity. HLH-2004-protocol may be considered in cases with poor clinical response or if disease progression is suspected. Besides, the distinction between sepsis and HLH is often difficult, being two acute hyperinflammatory reactions, potentially fatal without early recognition and with completely different management that is critical for a favorable outcome. A high index of clinical suspicion of measles-associated sHLH is required for early diagnosis and prompt treatment. Sen et al. had suggested that a serum ferritin >10 000 µg/L is strongly supportive of HLH [16]. Similarly, Machowicz et al. suggested that in the presence of hyperferritinemia, splenomegaly, marked cytopenias, hypofibrinogenemia, low CRP, characteristic cytokine profile, hypertriglyceridemia, the other HLH criteria should be assessed [17].

## Conclusions

Amidst a period of measles outbreaks across Europe due to suboptimum vaccination coverage, increased awareness of possible measles-induced sHLH, together with early recognition and initiation of appropriate treatment is crucial to prevent a cytokine storm progressing to multiorgan failure. More intensive efforts are essential to improve vaccination rates and control outbreaks leading to enhanced herd immunity. Such an approach is critical for the protection of unvaccinated young infants as well as immunocompromised patients who are at the highest risk of measles-related sHLH.
